# Enhancing innovative delivery in schools using design thinking

**DOI:** 10.12688/f1000research.72860.1

**Published:** 2021-09-15

**Authors:** Sharmini Gopinathan, Anisha Haveena Kaur, Kanesaraj Ramasamy, Murali Raman

**Affiliations:** 1 Faculty of Management, Multimedia University, Cyberjaya, Selangor, 63000, Malaysia; 2 Faculty of Computing & Informatics, Multimedia University, Cyberjaya, Selangor, 63000, Malaysia; 3 Research & Innovation, Asia Pacific University, Jalan Teknologi 5, Taman Teknologi Malaysia, 57000, Malaysia

**Keywords:** Design thinking, innovative teaching, 21st-century teaching, Malaysian schools, innovative delivery

## Abstract

The pandemic has created challenges in all sectors of the economy and education. Traditional teaching approaches seem futile in the new context, thus the need to constantly reinvent the delivery to meet the fast-paced changes in the education domain. Hence, Design Thinking (DT) is an alternative approach that might be useful in the given context. DT is known to be a human-centric approach to innovative problem-solving processes. DT could be employed in the delivery process to develop twenty-first-century skills and enhance creativity and innovation, in an attempt to identify alternative solutions. The study explores the role of design thinking (DT) mindset in innovative delivery among teachers. It enhances and facilitates innovative content delivery by leveraging creativity. The study targeted 131 teachers from primary and secondary schools in Malaysia. Data was collected through an online survey and was analyzed using SmartPLS to establish relationships between DT and Innovative Delivery in schools. The data was further analyzed to seek co-relations between the DT steps and the successful transformation of content delivery by teachers. The study established a framework for the application of design thinking for teachers as the primary support in developing activities for their students. The outcome of this study will help fill the gap towards creating an interesting method of delivery in schools and constantly innovating the method to suit the evolving generation. It provides an in-depth reason as to why students are not interested in the teacher's lessons which, in turn, affects their performance. This insight is crucial for the Ministry of Education and policymakers to enhance teachers’ ability to innovatively deliver content to students.

## Introduction

We are living in unprecedented times. The COVID-19 pandemic coupled with the continuous onslaught of digital technologies has acerbated the level of volatility, uncertainty, complexity, and ambiguity (VUCA), across various sectors. The education sector is not spared and remains a target for greater industrial shifts and repositioning in the context of remaining relevant. To this end, even schools are subject to changes—failure to innovate and offer the state of curriculum and pedagogy puts extreme pressure on students in their quest to become employable at later parts of life. As such, to ensure the education systems keep abreast with changes, strategic and practical shifts in the delivery of educational content is crucial. Failure to do so could result in longer term socioeconomic consequences.

In this paper, we posit that innovative delivery of educational content is required to ensure students can truly benefit from the learning outcomes set out in schools. Therefore, we propose precursors such as having empathy, rejuvenated thinking process, curriculum enhancement and gamification of lessons that can lead to innovative delivery of lessons. This includes examining the role of design thinking (DT) in generating innovative delivery of educational content.

There are various definitions of design thinking. According to Oxford Languages ^1^, the word ‘design’, a noun, refers to “a plan or drawing produced to show the look and function or workings of a building, garment, or other objects before it is made”. This definition implies that ‘design’ relates to any form of idea that is put forth either in the form of sketch, model, or better still as a full-blown prototype before the final product or model is developed. The second word, ‘thinking’, a noun, refers to “the process of considering or reasoning about something,” according to Oxford Languages ^2^. The keywords based on this definition are reasoning and process. DT therefore can be defined as a systematic or a structured approach to developing something, initially as a model or prototype, before a final version is built. We need to understand nevertheless that when people build or develop something, it is often done to solve a real-world problem. As such, DT is often defined as a systematic and structured approach to solving a problem based on design.

The inherent ideas beneath DT are not new. Nevertheless, DT presents an organised way of including innovative thinking and creativity in organisations. To be able to derive the inspirational values of DT, highly specific tools and techniques are used that are usually presented in a simplified manner. DT is able to solve problems using a user-centric collaborative method ^3^. Stanford University’s design school (dSchool) established a five-step DT process ^4^, which is summarised in Table 1.

**Table 1.** Phases of design thinking based on the understanding of Stanford School.

DT Phase	Brief Explanation
Empathy	The most vital step in DT. Design thinkers are given a design challenge to understand the emotional attachment and pain points of customers towards a problem or challenge. The findings from this step will naturally lead to the second step called ‘define’.
Define	This step requires design thinkers to focus on getting deep into the problem from the customer’s viewpoint. They will then spend some time to come up with specific perspectives. In addition, they will also suggest some game-changing propositions to the problem based on insights or intuitions.
Ideate	This step allows design thinkers to come up with as many ideas as they can in order to address the issue or problem which has been defined. Ideate is similar to brainstorming sessions.
Prototype	The step where ideas are translated into tangible manifestations. The prototype may not necessarily be a tangible product as it could also be a simulation, campaign, or mockup (this depends on the challenge at hand).
Test	This final step in DT emphasises on the importance of pitching the idea to indemnified target groups. This is followed by the gathering of feedback. The solution is either launched to the market or is reworked until it is deemed to be suitable for market launch.

DT when institutionalised as an inherent culture in schools, could lead to innovative teaching and learning processes. Specifically, DT has the potential to cultivate empathy, lead to a growth mindset (new thinking processes), and lead to curriculum enhancement in the form of gamified learning.

### Empathy

Empathy as a core of every DT project, one outcome of successful DT applications is the ability to encourage a culture that gives everybody a chance to express themselves freely. Empathy in the classroom context refers to the teacher-student interaction. Empathy shows the connection of what a teacher thinks or knows about their students and what they do to provide the necessary response to the students’ needs ^5^. This is also needed as teachers are the ones who arrange learning experiences for their students. Teachers are able to do so by providing feedback to their students. According to Mueller and Dweck ^6^, students who are praised and given feedback based on their efforts (instead of intelligence) are more likely to show an interest in mastery and tend to seek challenges when attempting to achieve their learning goals. These students can think out of the box as they are under the impression that their performances can be improved ^7^.

### Thinking process

Innovative and creative thinking is the product of DT’s ideation. The thinking process closely relates to the Growth Mindset Theory by Dweck ^8^. Students with a growing mindset tend to learn through persistence, failure, and different strategies. Additionally, students are able to overcome challenges given to them by practicing and using setbacks as a form of motivation. Implementation of innovative teaching strategies such as collaborative learning, using real-life problems to address issues, and experimentation also contribute to the thinking process ^9^. According to the Cambridge Learning Attributes Guide ^10^, the thinking process is a powerful tool which not only requires knowledge and understanding of a subject matter but also the students’ willingness to question it. By ensuring that students are provided with materials that enable them to challenge the subject matter they will be able to express their own understanding and opinions on it.

### Gamifying lessons

Prototyping in DT leads to a new form of learning, which in this context refers to gamification. Gamifying lessons enables teachers to establish a casual learning environment whereby students are able to challenge themselves via fun online games ^11^. According to Hakak *et al*. ^12^, students are given tasks or “missions” with varying levels of difficulty and they are required to complete them within a short time frame. They are also given the chance to repeat the “mission” if they fail to achieve the goal. This allows students to analyse and correct the mistakes made, which in turn encourages them to build a positive attitude towards learning ^13^. Eleftheria *et al*. ^14^ believe that the use of gamification provides students with a comprehensive understanding of the subject being taught and it increases their engagement and enjoyment in the learning process.

### Curriculum enhancement

Curriculum enhancement is the product of testing from DT. The materials provided in an enhanced curriculum should allow students to deeply reflect the topic at hand and provide them with the opportunities to make connections between other subjects and topics as well ^10^. Additionally, the curriculum should look beyond testing. Assessments in the form of evaluating students’ points of view and their observations are important as this shows the process of their progression ^15^. Teachers are also advised to consider implementing more group work and interactive lessons which builds on what students already know. From there, students are able to apply existing knowledge and add value to new knowledge.

As such, this paper aims to answer the following research questions:

1. Is there a relationship between DT and innovative delivery of teaching content in schools, specifically using the stage called empathy?
2. In addition to DT and empathy, what role does new thinking process, curriculum enhancement and gamification of lessons play towards similar aspirations?

Hence, the fulcrum of the study’s objectives is:

1. To determine if DT in the form of empathy leads to innovative delivery of curriculum
2. To examine if DT can lead to new forms of thinking processes and thus lead to innovative delivery of curriculum
3. To assess whether DT does lead to innovative delivery of curriculum, by promoting curriculum enhancement and gamified approach to teaching and learning.

## Methods

This research is purely quantitative whereby online survey was used as a means of data collection. Table 2 depicts the design elements used for this study. Questionnaires were carefully prepared with the anonymity of the respondents safe guarded ( See underlying data) ^16^. This was ensured as no personal data identifiers were collected. Additionally, an ethics approval was obtained before recruiting participants for the survey. From primary and secondary Malaysian schools, 200 teachers were invited to participate, however only 131 teachers responded. The items were adopted and adapted from various theories and previous studies conducted by Mueller and Dweck ^6^, Dweck ^8^, Hakak *et al*. ^12^, Eleftheria *et al*. ^14^ and Gipps ^15^. They were measured using the 5-point Likert scale, ranging from strongly disagree (1 points) to strongly agree (5 points). The odd Likert scale to give the survey respondents a choice to respond neutrally, was included. This was done to obtain evidence about a theme by adding a neutral response option for the respondents to select, should they refrain from selecting an answer from the two extreme choices. The scale offers five answer options. Table 2 depicts the research design components and their respective rationalizations.

**Table 2.** Research design elements.

Research Design Component	Description	Rationalisation
Nature of Study	Exploratory	The premise of this research is to determine whether design thinking leads to innovative delivery of lessons, especially in primary and secondary schools in Malaysia, as there is inadequate research in this particular domain of study.
Role of Theory	To test the theory	A deductive approach was employed for this study to test the hypothetical framework, namely the role of empathy, thinking process, curriculum enhancement and gamifying lessons in enhancing innovative delivery.
Sampling Process	Purposive sampling	A list of all primary and secondary public schools under the Ministry of Education Malaysia was attained by the researchers. The schools for this research were chosen based on Excel's RAND (random) function. The contact information of the teachers from the selected schools were attained from the National Union of the Teaching Profession (NUTP) as well as from the headmasters of those schools.
Data Collection Technique	Surveys	Due to the current COVID-19 outbreak, the questionnaire was prepared using Google Forms and was distributed to the primary and secondary public-school teachers via email, WhatsApp, and social media. A minimum of 129 respondents are required as per the G*Power analysis, 131 responses were collected at the end of the data collection period of one month. After data cleaning was conducted, there were no representation of teachers from the states of Perlis and Johore as well as the Federal Territory of Labuan. The teachers were not reachable/contactable due to the Movement Control Order (MCO) implemented by the government of Malaysia to curb the spread of COVID-19. Thus, there were no representatives from these states.
Researcher Interference	Minimal	There was minimal interference to the work nature and teacher activities by the researchers during the distribution and collection of questionnaires.

## Data analysis

The data for this study was analysed using the SmartPLS 3 software.

### Measurement model evaluation

**Figure 1.**
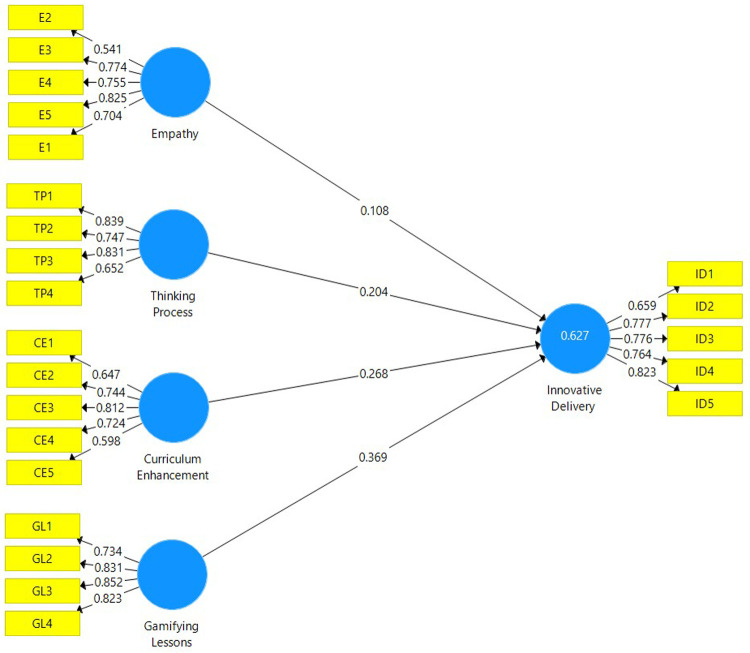
Measurement model depicting the latent variables and their respective indicators.

The measurement model evaluation is required to affirm the reliability and validity of the research model. The data attained from the questionnaires ( See underlying data) ^16^ were used to structure the measurement model of this study ( Figure 1)

Indicator reliability is assessed by ensuring that the factor loadings for each item is above 0.708. However, there is a satisfactory threshold whereby the values of each item do not necessarily have to be above 0.708. Table 3 affirms that the loadings for each item fall within the satisfactory value, thus indicator reliability is present. Internal consistency reliability is determined by the composite reliability (CR). As depicted in Table 2, the CR values for each construct are well above the 0.70 threshold, hence this affirms that the internal consistency reliability is satisfactory. Convergent validity is determined by the Average Variance Extracted (AVE). The AVE values for each construct must be above 0.50. The AVE for each construct in Table 2 is well above 0.50 and this signifies a satisfactory level of convergent validity for the study.

**Table 3.** Factor loadings, Average Variance Extracted and composite reliability of each construct.

Constructs	Items	Loadings	AVE	CR
Curriculum Enhancement	CE1	0.647	0.503	0.833
	CE2	0.744		
	CE3	0.812		
	CE4	0.724		
	CE4	0.598		
Empathy	E1	0.704	0.528	0.846
	E2	0.541		
	E3	0.774		
	E4	0.755		
	E5	0.825		
Gamifying Lessons	GL1	0.734	0.658	0.885
	GL2	0.831		
	GL3	0.852		
	GL4	0.823		
Thinking Process	TP1	0.839	0.595	0.853
	TP2	0.747		
	TP3	0.831		
	TP4	0.652		
Innovative Delivery	ID1	0.659	0.58	0.873
	ID2	0.777		
	ID3	0.776		
	ID4	0.764		
	ID5	0.823		

AVE: Average Variance Extracted; CR: Composite Reliability

Discriminant validity is evaluated according to the Fornell and Larcker criterion, whereby an item must show a stronger loading on its own construct when compared to other constructs. Table 4 affirms that each item has a stronger loading on its own construct, therefore, discriminant validity is fulfilled.

**Table 4.** Discriminant validity matrix.

Constructs	1	2	3	4	5
Curriculum Enhancement	0.709				
Empathy	0.623	0.726			
Gamifying Lessons	0.540	0.483	0.811		
Innovative Delivery	0.684	0.595	0.666	0.762	
Thinking Process	0.701	0.697	0.492	0.657	0.771

### Structural model evaluation

**Figure 2.**
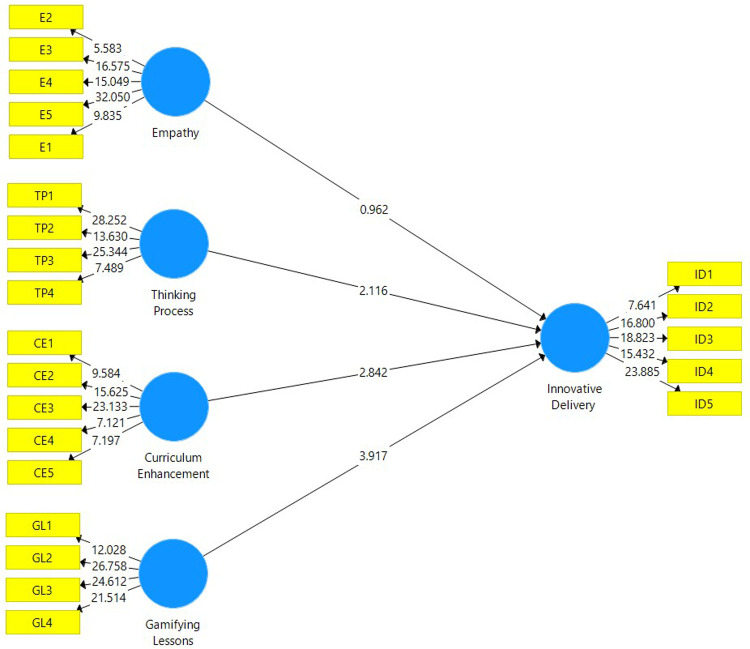
Structural model attained after evaluating the measurement model.

The structural model evaluation is conducted to determine whether the hypotheses are supported by the data attained from the analysis. The structural model depicted in Figure 2 is attained after a non-parametric bootstrapping using a sample of 5,000 was conducted. Before assessing the path coefficient of this study, the coefficient of determination (R ^2^) is explained. The value of R ^2^ for this study is 0.627, which falls under the moderate category. This means that 62.7% of the total variance in Innovative Delivery is explained by Empathy, Thinking Process, Curriculum Enhancement and Gamifying Lessons.

The path coefficient for this study is depicted in Table 5. For the beta value to make an impact to the research model, the value must be at least 0.1 whereas the t-statistic has to be greater than 1.645 at an alpha level of 0.05 in order for it to be significant. Table 3 confirms that curriculum enhancement, gamifying lessons and thinking process have a significant positive influence in enhancing innovative delivery. However, empathy does not have a significant positive influence in enhancing innovative delivery.

**Table 5.** Beta value, t-statistics, p-value, and hypothesis decision.

Constructs	Beta	T-Statistic	P-Value	Decision
Curriculum Enhancement --> Innovative Delivery	0.268	2.842	0.005	Supported
Empathy --> Innovative Delivery	0.108	0.962	0.336	Not Supported
Gamifying Lessons --> Innovative Delivery	0.396	3.917	0.000	Supported
Thinking Process --> Innovative Delivery	0.204	2.116	0.034	Supported

T-value significant at ≥1.645; P-value is significant at < 0.05

## Discussion and conclusion

The study showed that the thinking process, gamifying lessons, and curriculum enhancement have positive significance for innovative delivery. However, the variable empathy was not supported and did not show a positive significant relationship. The absence of empathy among teachers can affect the educational process adversely. Empathy is a method of associating with others that shows you can comprehend that they are encountering something significant, even though you may not understand precisely how it feels for them ^17^. Empathy is an essential advantage that can assist teachers by enhancing the driving factors on students’ behaviour. Thus, the link between teachers’ empathy and innovative teaching is essential and since the hypothesis for this study pertaining to empathy is not supported, it means that Malaysian teachers in predominantly primary and secondary schools lack empathy, which indirectly creates a large gap or power distance between students and the teachers themselves. This situation further enhances inability to deliver dry and uninteresting teaching and learning material which leads to hatred and disinterest among students in the subject. The current pandemic has made students more productive, independent, and proactive in being responsible for their learning. As such, the overall results of the Sijil Pelajaran Malaysia (SPM) 2020 or the Malaysian Certificate of Education, which is a national examination taken by all fifth-form secondary school students in Malaysia. have shown a sharp rise in passing and grades as compared to previous years ^18^. This also indicates that teachers are no longer seen as mere content providers and knowledge givers, but as facilitators and support during difficult times. The lack of empathy among teachers must be addressed if teachers are exposed to DT workshops during their formal training and periodically as part of their learning development programme. The outcome of this study shows the aspects which need to be addressed by the Ministry of Education as well as the teachers in Malaysia. In addition, the outcome of this study may also assist in producing teachers who are well rounded in terms of mastering various teaching skills.

## Ethics approval

The Research Ethics Committee (REC) of Multimedia University has granted the ethics approval for this research with the approval number EA2232021. 

## Data availability

### Underlying data

Figshare: Enhancing innovative delivery in schools using design thinking

DOI: 10.6084/m9.figshare.14871879 ^16^.

This project contains the following underlying data:

Data file 1. The data attained from the questionnaire. This file is to be opened using the SPSS software.

Data file 2. Questionnaire used for this research.

Data are available under the terms of the Creative Commons Zero "No rights reserved" data waiver (CC0 1.0 Public domain dedication).
