# Supplementary material for: Enhancing innovative delivery in schools using design thinking
Source: F1000Res. 2022 Jan 10;10:927. Originally published 2021 Sep 15. [Version 3] doi: 10.12688/f1000research.72860.3 (PMC8753572; doi:10.12688/f1000research.72860.3)
Supplement: Peer review history

# Peer review: Reviewer response for version 3

**DOI:** 10.5256/f1000research.114839.r119249

**Published:** 2022-01-11

**Authors:** Paulo SImeão Carvalho

**Affiliations:** 1 Physics and Astronomy Department, Science Education Unit, Faculty of Sciences, University of Porto, Porto, Portugal

I have no further comments to make concerning this paper. In my point of view, it is now approved for indexing.

Is the work clearly and accurately presented and does it cite the current literature?

Yes

If applicable, is the statistical analysis and its interpretation appropriate?

I cannot comment. A qualified statistician is required.

Are all the source data underlying the results available to ensure full reproducibility?

Yes

Is the study design appropriate and is the work technically sound?

Yes

Are the conclusions drawn adequately supported by the results?

Partly

Are sufficient details of methods and analysis provided to allow replication by others?

Yes

Reviewer Expertise:

Physics education

I confirm that I have read this submission and believe that I have an appropriate level of expertise to confirm that it is of an acceptable scientific standard.

# Peer review: Reviewer response for version 2

**DOI:** 10.5256/f1000research.79753.r101527

**Published:** 2021-12-15

**Authors:** Paulo SImeão Carvalho

**Affiliations:** 1 Physics and Astronomy Department, Science Education Unit, Faculty of Sciences, University of Porto, Porto, Portugal

The authors improved the content of the paper a lot by making adequate changes to the text.

 However, I still find some items that must be clarified (page numbers refer to the pdf version of the article).

- Page 5, right column, line 20: still concerning the statement “alpha level of 0.05”. If this information concerns a significance level as the authors claim, then the legend of Table 5 should mention it. Moreover, this alpha level is not introduced or explained in the text, so additional information is lacking.
- Page 8, left column, line 3-6: although the sentence “Students don’t feel attracted to the old method of teaching, as such based on the lack of empathy and inadequacy of skills to innovate delivery make it even worse” has been introduced to improve the writing. it sounds strange and confusing. Authors must rewrite it carefully to clarify what they want to say.

Minor changes:

- Abstract, line 23: instead of “The study showed” I suggest “It shows”, because the words “The study” are repeated twice very closely.
- Page 8, left column, line 6-7: the sentence “Ministry of Education Malaysia” should be changed to Malaysian Ministry of Education.

In conclusion:

 I believe the paper is interesting for discussion about the influence of several parameters to innovative delivery in schools, taking for a particular example primary and secondary teachers of Malaysia.

 I think some minor but important issues should be clarified by the authors, before I can approve the paper for indexing.

Is the work clearly and accurately presented and does it cite the current literature?

Yes

If applicable, is the statistical analysis and its interpretation appropriate?

I cannot comment. A qualified statistician is required.

Are all the source data underlying the results available to ensure full reproducibility?

Yes

Is the study design appropriate and is the work technically sound?

Yes

Are the conclusions drawn adequately supported by the results?

Partly

Are sufficient details of methods and analysis provided to allow replication by others?

Yes

Reviewer Expertise:

NA

I confirm that I have read this submission and believe that I have an appropriate level of expertise to confirm that it is of an acceptable scientific standard, however I have significant reservations, as outlined above.

# Response

**Published:** 2021-12-17

**Authors:** Sharmini Gopinathan

**Affiliations:** Multimedia University, Malaysia

(Page numbers refer to the pdf version of the article)

 1. Page 5, right column, line 20: still concerning the statement “alpha level of 0.05”. If this information concerns a significance level as the authors claim, then the legend of Table 5 should mention it. Moreover, this alpha level is not introduced or explained in the text, so additional information is lacking.

*- Since the alpha level is not introduced/explained in the text, we have decided to remove it. The statement has been changed to “the t-statistic has to be greater than 1.645” (in order to maintain consistency just like the statement mentioning the beta value).*

**

 2. Page 8, left column, line 3-6: although the sentence “Students don’t feel attracted to the old method of teaching, as such based on the lack of empathy and inadequacy of skills to innovate delivery make it even worse” has been introduced to improve the writing. it sounds strange and confusing. Authors must rewrite it carefully to clarify what they want to say.

*- We meant to say that the lack of empathy from the teachers who participated in the survey affects the learning experience of the students as well (apart from it indirectly creating a large gap/power distance between students and teachers). Thus, we have reworded it to say:*

*“Thus, the link between teachers’ empathy and innovative teaching is essential and since the hypothesis for this study pertaining to empathy is not supported, it means that the Malaysian teachers who participated in this study predominantly lack empathy, which indirectly creates a large gap or power distance between students and the teachers themselves. Additionally, it affects the learning experience of the students as well.”*

 3. Abstract, line 23: instead of “The study showed” I suggest “It shows”, because the words “The study” are repeated twice very closely.

*- We have amended it, as recommended.*

**

 4. Page 8, left column, line 6-7: the sentence “Ministry of Education Malaysia” should be changed to Malaysian Ministry of Education.

*- We have amended it, as recommended.*

# Peer review: Reviewer response for version 2

**DOI:** 10.5256/f1000research.79753.r101528

**Published:** 2021-12-03

**Authors:** Achmad Samsudin

**Affiliations:** 1 Department of Physics Education, Universitas Pendidikan Indonesia, Bandung, Indonesia

It's according to the suggestion.

Is the work clearly and accurately presented and does it cite the current literature?

Yes

If applicable, is the statistical analysis and its interpretation appropriate?

Yes

Are all the source data underlying the results available to ensure full reproducibility?

Yes

Is the study design appropriate and is the work technically sound?

Yes

Are the conclusions drawn adequately supported by the results?

Yes

Are sufficient details of methods and analysis provided to allow replication by others?

Partly

Reviewer Expertise:

Science education, conception, conceptual change, misconception in physics, development of models, media, and instruments

I confirm that I have read this submission and believe that I have an appropriate level of expertise to confirm that it is of an acceptable scientific standard.

# Peer review: Reviewer response for version 1

**DOI:** 10.5256/f1000research.76468.r95938

**Published:** 2021-11-15

**Authors:** Achmad Samsudin

**Affiliations:** 1 Department of Physics Education, Universitas Pendidikan Indonesia, Bandung, Indonesia

Abstract

 Explain in the abstract section the research questions used and the result.

 Describe the number of teachers for each school (primary and secondary schools).

 Describe what survey instruments were used? Open/close-ended, how many questions, using a Likert scale or something like that, etc.

Introduction

 Is there any previous research that discusses something similar? And what is the novelty of this research? Explain in this section.

 Add citations for your opinion. Is it necessary to use innovative delivery to benefit from learning outcomes?

 In the fulcrum of the study’s objectives, this section should serve as an answer to a research question, but why are the numbers different? It should only be the same number of research questions.

Methods

 Put the design in the abstract.

 For respondents, describe the number of teachers for each school (primary and secondary schools).

 Explain why the SmartPLS 3 software is used in this study. If necessary add citations to strengthen it.

 Note in Table 1, there are 5 DT Phases (of course this section is an indicator in this study). However, why in Figure 1 do only 4 of the 5 phases exist? The missing piece is "Prototype", why is that?

 Same for Figure 2, why do only 4 of the 5 phases exist? The missing piece is "Prototype", why is that?

Is the work clearly and accurately presented and does it cite the current literature?

Yes

If applicable, is the statistical analysis and its interpretation appropriate?

Yes

Are all the source data underlying the results available to ensure full reproducibility?

Yes

Is the study design appropriate and is the work technically sound?

Yes

Are the conclusions drawn adequately supported by the results?

Yes

Are sufficient details of methods and analysis provided to allow replication by others?

Partly

Reviewer Expertise:

Science education, conception, conceptual change, misconception in physics, development of models, media, and instruments

I confirm that I have read this submission and believe that I have an appropriate level of expertise to confirm that it is of an acceptable scientific standard, however I have significant reservations, as outlined above.

# Response

**Published:** 2021-11-23

**Authors:** Sharmini Gopinathan

**Affiliations:** Multimedia University, Malaysia

**Abstract**

 1. Explain in the abstract section the research questions used and the result.

*- Included in the abstract, as recommended.*

 2. Describe the number of teachers for each school (primary and secondary schools).

*- The number of teachers for each school has been added, as recommended. 61 of the respondents are primary school teachers and 70 of the respondents are secondary school teachers.*

 3. Describe what survey instruments were used? Open/close-ended, how many questions, using a Likert scale or something like that, etc.

*- The description of the instrument has been added. Questionnaire was used and there were 23 close-ended questions. A 5-point Likert scale was used.*

**Introduction**

 1. Is there any previous research that discusses something similar? And what is the novelty of this research? Explain in this section. Add citations for your opinion.

*- We have included an explanation about DT in the education context, specifically in schools, whereby there is limited empirical research.*

 2. Is it necessary to use innovative delivery to benefit from learning outcomes?

*- Yes, this is much needed in the Malaysian context, especially with the sudden transition from face-to-face teaching to online teaching due to the pandemic. Teachers and schools are finding new and innovative ways to deliver their learning outcomes.*

 3. In the fulcrum of the study’s objectives, this section should serve as an answer to a research question, but why are the numbers different? It should only be the same number of research questions.

*- We have amended the research objectives to suit the research questions. The research objectives are as follows:*

- ***1.*** *To determine if DT in the form of empathy leads to innovative delivery of curriculum.*
- ***2.*** *To examine if DT can lead to new forms of thinking processes, gamified lessons and curriculum enhancement, thus leading to innovative delivery of curriculum.*

**Methods**

 1. Put the design in the abstract.

*- Added. Included the use of questionnaire, number of questions and the Likert scale used.*

 2. For respondents, describe the number of teachers for each school (primary and secondary schools).

*- The number of teachers for each school have been added, as recommended. 61 of the respondents are primary school teachers and 70 of the respondents are secondary school teachers.*

 3. Explain why the SmartPLS 3 software is used in this study. If necessary add citations to strengthen it.

*- SmartPLS 3 software was used as it is able to analyse inter-relationships between variables, whereby single/multiple regressions can be stated (Hair et al., 2011).*

 4. Note in Table 1, there are 5 DT Phases (of course this section is an indicator in this study). However, why in Figure 1 do only 4 of the 5 phases exist? The missing piece is "Prototype", why is that? Same for Figure 2, why do only 4 of the 5 phases exist? The missing piece is "Prototype", why is that?

*- As per the Introduction section, “Prototype” falls under the sub-section “Gamifying Lessons”. We have described it as such: “Prototyping in DT leads to a new form of learning, which in this context refers to gamification”.*

*References*

 Hair, J. F., Ringle, C. M., & Sarstedt, M. (2011). PLS-SEM: Indeed a silver bullet. *Journal of Marketing theory and Practice*, *19*(2), 139-152. DOI: 10.2753/MTP1069-6679190202

# Peer review: Reviewer response for version 1

**DOI:** 10.5256/f1000research.76468.r95936

**Published:** 2021-10-26

**Authors:** Paulo SImeão Carvalho

**Affiliations:** 1 Physics and Astronomy Department, Science Education Unit, Faculty of Sciences, University of Porto, Porto, Portugal

The paper describes the role of design thinking that can lead to innovative teaching among teachers. In particular, it presents a study that seeks how several variables such as empathy, thinking process, gamifying lessons and curriculum enhancement, may have positive significance for innovative delivery. The study targeted 131 teachers from primary and secondary schools in Malaysia.

 I find this paper has great interest for teachers, although it concerns only Malaysian ones. The case cannot be extrapolated to other countries, but undoubtedly points out to real educational problems that should be seen very seriously by decision makers.

 Concerning the sample, it is not clear how many of those 131 teachers were from primary school and secondary school. As far as I know, the teaching goals, the contact hours and the involvement of teachers with their students may not be exactly the same for both categories of teacher, therefore the authors should provide separate analysis for both samples – even if, at the end, they conclude the results are equivalent.

 There are some issues that could be clarified by the authors (page numbers refer to the pdf version):

1. In page 3, line 4 (Introduction), left column, the acronym VUCA is not used in the paper. I suggest to remove it, unless there is a specific reason to keep it.
2. Page 4, line 4, left column: I would say that not only collaborative, but also cooperative learning should be recommended, as the latter involves students in the responsibility for finding solutions to the problems.
3. Page 5, line 13, right column: instead of “the loadings for each item fall within the satisfactory value”, I suggest it should be added "for the majority of items" because as the authors recognize, there are some items that do not fulfill that condition.
4. Page 4, figure 1, empathy variables: why is the sequence, from top to bottom, different from the other design components? i.e., why not E1; E2; E3; E4; E5 ? The same comment applies to Figure 2.
5. Page 6, line 12, right column: alpha value, or p-value?
6. Page 6, line 32-33, right column: the sentence “Malaysian teachers in predominantly primary and secondary schools lack empathy” is somehow exaggerated. This study is based only in 131 responses, which do not represent, in any way, Malaysian teachers. The authors cannot make such a generalization. This sentence MUST be reformulated to the local validity of this study.
7. Page 6, line 36-38, right column: I don't see a direct correlation between the results obtained and the conclusion that “This situation further enhances inability to deliver dry and uninteresting teaching and learning material”. The authors should discuss better how they figure out such a correlation derived from their definition of Empathy. Their extrapolation is very broad and I don't think it has support in this investigation. Perhaps the authors want to rephrase it as a possibility that might occur, but not as an affirmative statement.
8. Page 6, table 2, line 5: item “CE4” seems to be “CE5”.
9. Finally, the reference list seems adequate to the study reported, and the English is of high quality.

In conclusion:

 I believe the paper is interesting for discussion about the influence of several parameters to innovative delivery in schools, taking for a particular example primary and secondary teachers in Malaysia. However, some topics could be clarified and generalizations should be avoided because the study is not vast enough.

 So, I recommend this paper for indexing, but the authors must correct some errors and clarify the items mentioned first.

Is the work clearly and accurately presented and does it cite the current literature?

Yes

If applicable, is the statistical analysis and its interpretation appropriate?

I cannot comment. A qualified statistician is required.

Are all the source data underlying the results available to ensure full reproducibility?

Yes

Is the study design appropriate and is the work technically sound?

Yes

Are the conclusions drawn adequately supported by the results?

Partly

Are sufficient details of methods and analysis provided to allow replication by others?

Yes

Reviewer Expertise:

Physics education; teacher training in phusics; digital educational resources; experimental work

I confirm that I have read this submission and believe that I have an appropriate level of expertise to confirm that it is of an acceptable scientific standard, however I have significant reservations, as outlined above.

# Response

**Published:** 2021-11-23

**Authors:** Sharmini Gopinathan

**Affiliations:** Multimedia University, Malaysia

(Page numbers refer to the pdf version of the article)

 1. In page 3, line 4 (Introduction), left column, the acronym VUCA is not used in the paper. I suggest to remove it, unless there is a specific reason to keep it.

*-The acronym VUCA has been removed as it is not used in the paper.*

**

 2. Page 4, line 4, left column: I would say that not only collaborative, but also cooperative learning should be recommended, as the latter involves students in the responsibility for finding solutions to the problems.

*- A very good recommendation by the reviewer. However, as the statement was attained from a citation, we are not able to add it in the sentence as it will not reflect what was originally interpreted/meant by the author (of the citation). We shall take note of cooperative learning for future studies.*

**

 3. Page 5, line 13, right column: instead of “the loadings for each item fall within the satisfactory value”, I suggest it should be added "for the majority of items" because as the authors recognize, there are some items that do not fulfil that condition.

*- “Each item” has been replaced with “for the majority of items”, as recommended.*

**

 4. Page 4, figure 1, empathy variables: why is the sequence, from top to bottom, different from the other design components? i.e., why not E1; E2; E3; E4; E5 ? The same comment applies to Figure 2.

*- The sequence was automatically generated by the SmartPLS 3.0 software. As the figures were directly taken from the (measurement and structural) models generated by the software, we have decided to not alter it and retain the sequence the way it is.*

**

*5. *Page 6, line 12, right column: alpha value, or p-value?

*- Alpha value (significance level).*

**

 6. Page 6, line 32-33, right column: the sentence “Malaysian teachers in predominantly primary and secondary schools lack empathy” is somehow exaggerated. This study is based only in 131 responses, which do not represent, in any way, Malaysian teachers. The authors cannot make such a generalization. This sentence MUST be reformulated to the local validity of this study.

*- The sentence has been changed to “* *Thus, the link between teachers’ empathy and innovative teaching is essential and since the hypothesis for this study pertaining to empathy is not supported, it means that the Malaysian teachers who participated in this study predominantly lack empathy, which indirectly creates a large gap or power distance between students and the teachers themselves”* *. This was done to avoid generalisation of the whole population.*

**

 7. Page 6, line 36-38, right column: I don't see a direct correlation between the results obtained and the conclusion that “This situation further enhances inability to deliver dry and uninteresting teaching and learning material”. The authors should discuss better how they figure out such a correlation derived from their definition of Empathy. Their extrapolation is very broad and I don't think it has support in this investigation. Perhaps the authors want to rephrase it as a possibility that might occur, but not as an affirmative statement.

*- Rephrased as suggested by the reviewer.*

**

 8. Page 6, table 2, line 5: item “CE4” seems to be “CE5”.

*- CE4 has been changed to CE5.*

**

 9. Finally, the reference list seems adequate to the study reported, and the English is of high quality.

*- Thank you.*

 10. Concerning the sample, it is not clear how many of those 131 teachers were from primary school and secondary school. As far as I know, the teaching goals, the contact hours and the involvement of teachers with their students may not be exactly the same for both categories of teacher, therefore the authors should provide separate analysis for both samples – even if, at the end, they conclude the results are equivalent.

*- 61 of the respondents are primary school teachers and the remaining 70 respondents are secondary school teachers. As this is not a comparison study between primary and secondary school teachers, we have decided to retain the current analysis as is it.*
